# Functional requirements for homeostatic inhibitory plasticity in recurrent networks

**DOI:** 10.1186/1471-2202-16-S1-P234

**Published:** 2015-12-18

**Authors:** Owen Mackwood, Henning Sprekeler

**Affiliations:** 1 Technische Universität Berlin, 10587 Berlin, Germany; 2 Bernstein Center for Computational Neuroscience, 10115 Berlin, Germany

## 

Homeostatic regulation of neuronal firing rates has been reliably observed in response to chronic manipulation of neural activity. From such experiments a variety of putative homeostatic mechanisms have been reported, including compensatory changes for several synapse types [1]. Unfortunately, it is sometimes unclear whether these synaptic changes occur in response to persistent rate deviations in individual neurons (local control) or in larger populations (global control). One variant of the former type is the recently proposed Hebbian learning rule of Vogels et al., which modifies inhibitory-to-excitatory synapses to maintain a target rate in each postsynaptic cell [2]. Since this rule asserts its control by changing inhibition, one might expect that in recurrent networks, similar synaptic plasticity rules elsewhere within the inhibitory feedback loop should be capable of comparable homeostatic control. Using simulations of locally connected recurrent networks, we investigate a plasticity rule that changes excitatory-to-inhibitory synapses such that presynaptic excitatory activity should remain at a target rate. We show that if the rule attempts to control excitatory neurons individually, it induces competition between those cells. For a Hebbian rule, this effect results in most of the network being driven to quiescence, whereas with rule dependent only upon presynaptic rates the competition in less pronounced. In either case, this competition is a consequence of having a local sensor (measuring the rate of a single cell), but actuators (synapses) with non-local effect -- synaptic changes triggered by an individual excitatory cell also affect other excitatory cells due to the "fanout" in recurrent connectivity. Said competition can be detected in the spontaneous firing rates of the population, which become sparser over time. If instead the synaptic learning rule attempts to control the average firing rate of neurons in a sufficiently large region, this competitive effect is mitigated. Our results suggest that an apparently homeostatic rule can lead to competition, whose degree depends upon the spatial specificity of its firing rate sensor and the spatial range of its effect on the network (the effector). This interaction between sensor and effector locality in homeostatic rate control implies that further insights into the synaptic mechanisms of homeostasis could be gained by studying neuronal responses to localized experimental manipulations.

## References

[B1] Turrigiano G Too Many Cooks? Intrinsic and Synaptic Homeostatic Mechanisms in Cortical Circuit Refinement Annu Rev Neurosci 2011 34 89 103 21438687 10.1146/annurev-neuro-060909-153238

[B2] Vogels TP Sprekeler H Zenke F Clopath C Gerstner W Inhibitory plasticity balances excitation and inhibition in sensory pathways and memory networks Science 2011 334 6062 1569 1573 22075724 10.1126/science.1211095

